# Efficacy and Safety of Endovascular Treatment versus Intravenous Thrombolysis for Acute Ischemic Stroke: A Meta-Analysis of Randomized Controlled Trials

**DOI:** 10.1371/journal.pone.0077849

**Published:** 2013-10-31

**Authors:** Chao Lin, Nan Li, Kang Wang, Xin Zhao, Bai-Qiang Li, Lei Sun, Yi-Xing Lin, Jie-Mei Fan, Miao Zhang, Hai-Chen Sun

**Affiliations:** 1 Department of Neurosurgery, Jinling Hospital, Nanjing University, Nanjing, China; 2 Department of Urology, Gulou Hospital, Nanjing University, Nanjing, China; University of Glasgow, United Kingdom

## Abstract

**Background and Purpose:**

Although endovascular therapy (ET) is increasingly used in patients with moderate to severe acute ischemic stroke, its efficacy and safety remains controversial. We performed a meta-analysis aiming to compare the benefits and safety of endovascular treatment and intravenous thrombolysis in the treatment of acute ischemic stroke.

**Methods:**

We systematically searched PubMed, Embase, Science direct and Springer unitil July, 2013. The primary outcomes included good outcome (mRS ≤ 2) and excellent outcome (mRS ≤ 1) at 90 days or at trial end point. Secondary outcomes were occurrence of symptomatic hemorrhage and all-cause mortality.

**Results:**

Using a prespecified search strategy, 5 RCTs with 1106 patients comparing ET and intravenous thrombolysis (IVT) were included in the meta-analysis. ET and IVT were associated with similar good (43.06% vs 41.78%; OR=1.14; 95% CI, 0.77 to 1.69; P=0.52;) and excellent (30.43% vs 30.42%; OR=1.05; 95% CI, 0.80 to 1.38; P=0.72;) outcome. For additional end points, ET was not associated with increased occurrence of symptomatic hemorrhage (6.25% vs. 6.22%; OR=1.03; 95% CI, 0.62 to 1.69; P=0.91;), or all-cause mortality (18.45% vs. 17.35%; OR=1.00; 95% CI, 0.73 to 1.39; P=0.99;).

**Conclusions:**

Formal meta-analysis indicates that there are similar safety outcomes and functional independence with endovascular therapy and intravenous thrombolysis for acute ischemic stroke.

## Introduction

Stroke is one of the leading causes of mortality and morbidity in the world [1][2]. More than 80% of all incident strokes are of ischemic type, caused by an occluding thrombus of a cerebral artery [3]. Intravenous recombinant tissue plasminogen activator (t-PA) is the standard treatment for acute ischemic stroke (AIS), but its clinical effectiveness is critically time-dependent [4],[5],[6]. Because of a relatively short therapeutic time window (<4.5 hours) after symptom onset, few patients with acute ischemic stroke meet current eligibility criteria for the use of intravenous t-PA [5]. Endovascular treatment has been used for many years. As compared with control group, endovascular treatment is associated with a higher probability of recanalization [7],[8]. However, the previous related studies tended to show different results when it compared with intravenous thrombolysis [9],[10][11][12]. Recommendations based on the results of individual trials may be misleading, and the previous review include only two small-scale (81 patients) studies [13]. We conducted this meta-analysis of all Randomized Controlled Trials (RCTs) to assess the efficacy and safety of endovascular treatment in patients with acute ischemic stroke as comparaed with intravenous thrombolysis.

## Methods

### Search Strategy

A systematic literature search up to July 2013 was performed in PubMed, Embase, Science direct and Springer to identify relevant studies without language restriction. The electronic search strategy included the terms (“endovascular treatment” OR “intra-arterial” OR “intravenous” OR “fibrinolysis” OR “thrombolysis” ) AND (“ischemic stroke” OR “brain infarct”) combined with “randomized controlled trial”. The titles and abstracts were scanned to exclude any clearly irrelevant studies. The full texts of the remaining articles were read to determine whether they contained information on the topic of interest. Furthermore, to find any additional published studies, a manual search was performed by checking all the references of original reports. In addition, we reviewed the cited lists of eligible trials by Google Scholar to ensure that all appropriate studies were included. All searches were conducted independently by 2 authors (CL and NL). The results were compared, and any questions or discrepancies were resolved through iteration and consensus.

### Selection Criteria

To be eligible, studies had to fulfill the following 4 inclusion criteria: (1) comparative study; (2) the study population consisted of patients with acute ischemic stroke; (3) reports comparaed endovascular treatment with intravenous thrombolysis; (4) and study was an RCT. Studies without data on comparison group were excluded from the study.

### Outcome Measures

The primary outcome chosen were good (modified Rankin Scale (mRS) score 0 to 2 or nearest equivalent) and excellent (mRS score 0 to 1 or nearest equivalent) clinical functional outcomes at 90 days or at trial end point. Secondary outcomes were: (1) occurrence of Symptomatic hemorrhage after treatment; and (2) all-cause mortality at the end of follow-up.

### Selection and Data Extraction

Information from studies was extracted independently by 2 researchers (CL and NL), with disagreements resolved by consensus. The following data were collected: the first author’s last name, year of publication, study design, study participants mean age and gender, sample size (cases and controls or cohort size), stroke severity (measured with the National Institutes of Health Stroke Scale, NIHSS), time window, intervention and follow-up days (Table 1). Selected RCTs were critically appraised using the Jadad scale, which scores studies’ description of randomization (2 points), blinding (2 points) and attrition information (1 point) [14].

**Table 1:** Characteristics of studies included in Meta-Analysis.

Source	study design	NO.	age, y	Sex (male)	NIHSS ( median)	Intervention	Time window (mean)	follow-up	Jadad score	Allocation concealment
Broderick 2013	RCT	656	18-82	340(51.83%)	ET 17,IVT 16	ET after IV t-PA versus IV t-PA	ET 5 h,IVT 3 h	90 days	5	Yes
Ciccone 2013	RCT	362	18-80	209(57.73%)	ET 13,IVT 13	ET versus IV t-PA	ET 3.75 h,IVT 2.75 h	90 days	5	Yes
Ciccone 2010	RCT	54	18-80	42(77.78%)	IAT 17,IVT 16	IA alteplase versus IV alteplase	IAT3.15 h,IVT 2.35 h	90 days	5	Yes
Sen 2009	RCT	7	68±16	5(71.43%)	16	IAt-PA versus IV t-PA	within 3 h	90 days	4*	Inadequate
Ducrocq 2005	RCT	27	18-79	21(77.78%)	Mean SSS:IAT 20.8,IVT 19.6	IA urokinase versus IV urokinase	IAT: 5.24h, IVT: 4.16h	90 days	4*	Yes

* Randomization method was not described.

NIHSS, National Institutes of Health Stroke Scale; RCT, Randomized Controlled Trial; ET, Endovascular Treatment; IAT, intra-arterial thrombolysis; IVT, intravenous thrombolysis; IA, intra-arterial; IV, intravenous; SSS, Scandinavian Stroke Scale; MCA, middle cerebral artery; NG, not give; h, hour; ITT, intention-to-treat.

### Quality Assessment and Statistical Analyses

We analysed dichotomous outcomes extracted from individual studies to compute individual study odds ratios (ORs) with 95% confidence intervals (CIs) and estimate the pooled Mantel-Haenszel (M-H) OR.

Heterogeneity of treatment effects between studies was investigated statistically by the heterogeneity I^2^ statistic. Cochran Q P values <0.1 and I^2^ ≥25% were considered as indicating significant heterogeneity [15]. When significant heterogeneity was absent, the fixed-effects model was employed; otherwise, the random-effects model was used.

A *P* value <0.05 was considered statistically significant. The publication bias was assessed through funnel plots. Sensitivity analyses were conducted to determine the influence of statistical models (the fixed-effects model and the random-effects model) on effect size. All statistical tests were performed by using the RevMan 5.1 software (Nordic Cochrane Center, Copenhagen, Denmark) and STATA 11.0 software (Stata Corporation, College Station, TX). The meta-analysis was performed in compliance with the Preferred Reporting Items for Systematic Reviews and Meta-Analyses (PRISMA) statement [16],[17].

## Results

### Characteristics of Selected Studies

**Figure 1:**
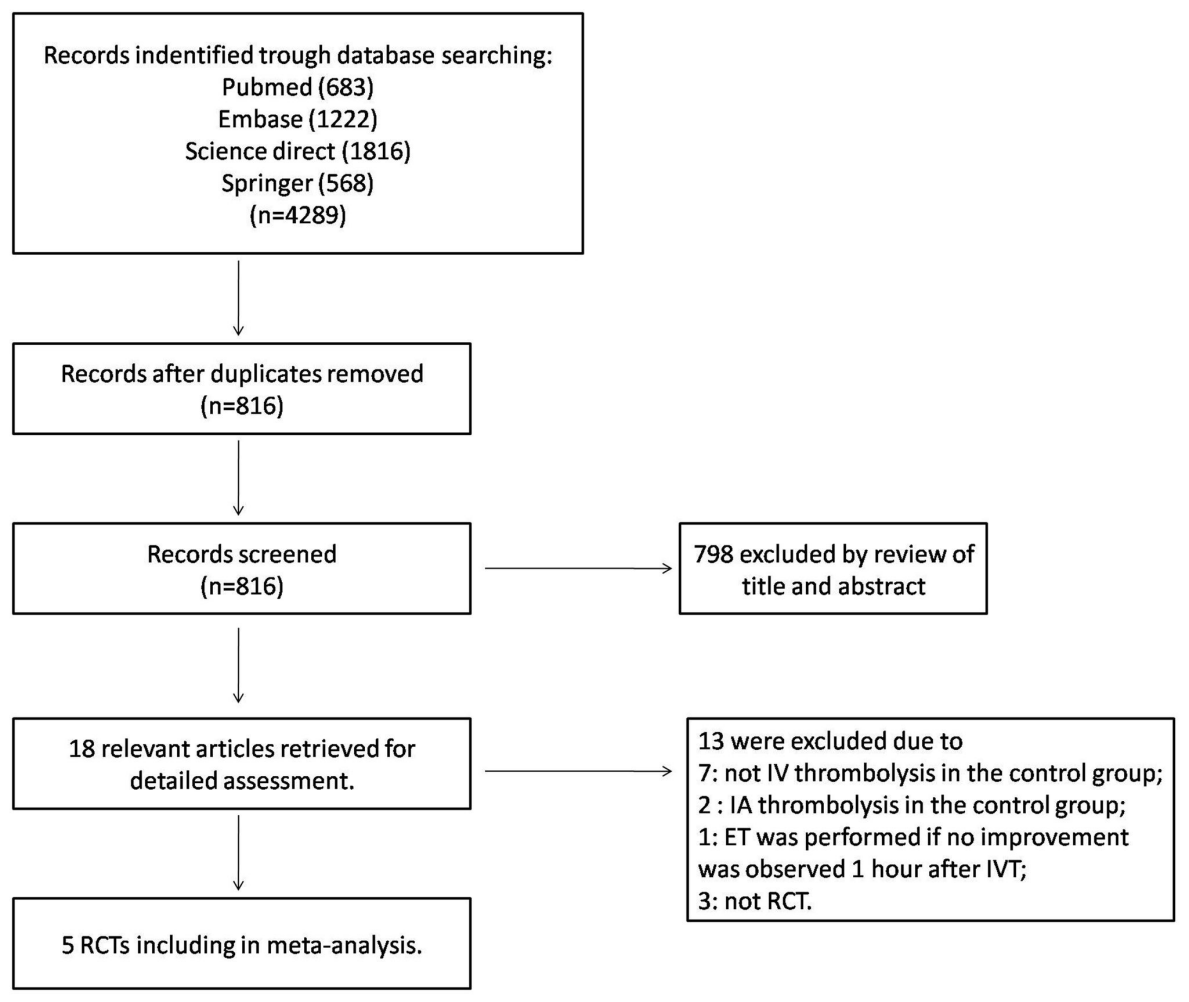
The flowchart shows the selection of studies for meta-analysis.

Our initial search strategy retrieved a total of 4289 citations. After screening of titles and abstracts, 14 eligible articles were selected. We identified 14 potentially relevant articles concerning endovascular therapy and intravenous thrombolysis in relation to acute ischemic stroke. Finally, 5 articles were included in this meta-analysis (Figure 1). 

All trials compared endovascular therapy with standard intravenous thrombolysis. All studies were described as RCT. These 5 RCTs included a total of 656 (59.31%) patients with endovascular therapy, and 450 with intravenous thrombolysis.

The study design, quality, and baseline characteristics of these studies are shown in Table 1. All RCTs were of high methodological quality with a satisfying Jadad score. The number of participants ranged from 7 to 656. Baseline median or mean National Institutes of Health Stroke Scale scores ranged from 13 to 17. In only one trial, ET was confounded by IVT [9]. The follow-up of all trials was 90 days.

### Primary Outcomes

#### Good outcome (modified Rankin Scale 0-2) and excellent outcome (modified Rankin Scale 0-1)

**Figure 2:**
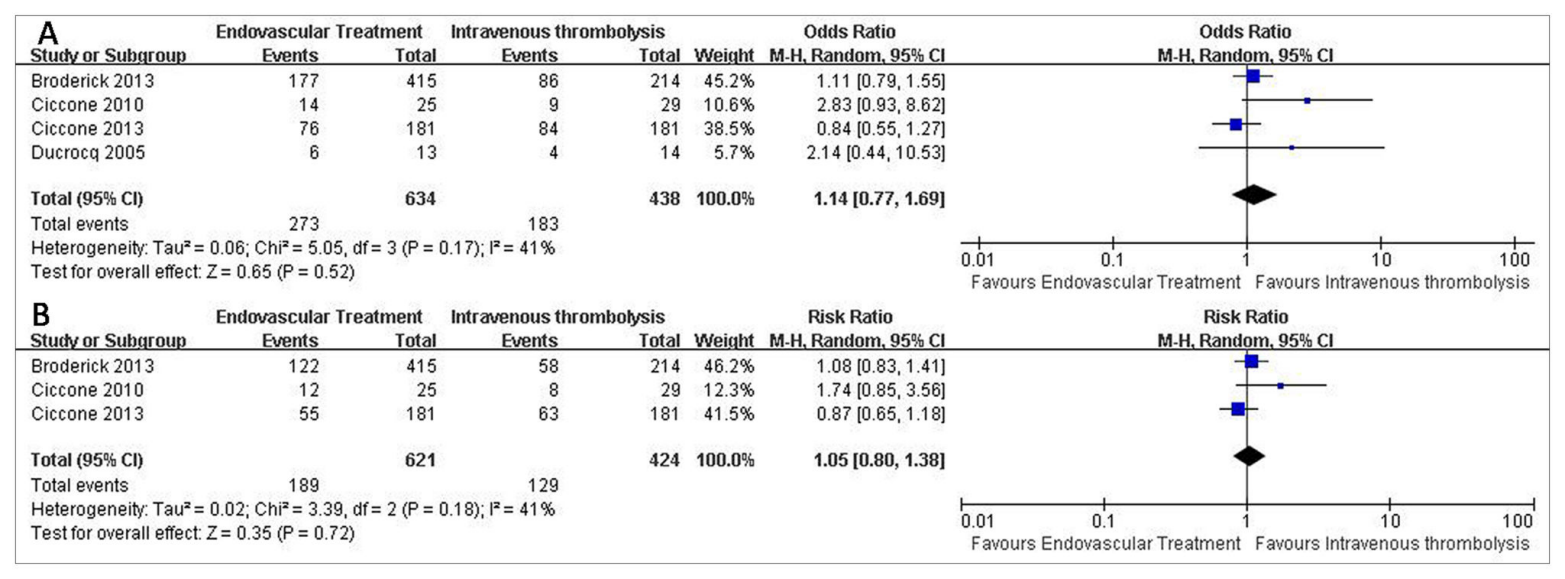
Primary Outcomes of patients treated with Endovascular Therapy and Intravenous Thrombolysis after acute ischemic stroke. (A) Forest plot of RR and 95% CI for the occurrence of good outcome in patients assigned to Endovascular Therapy and Intravenous Thrombolysis. (B) Forest plot of RR and 95% CI for the occurrence of excellent outcome in patients assigned to Endovascular Therapy and Intravenous Thrombolysis.

Four studies presented information on good outcome at follow-up (Figure 2A), and three on excellent outcome (Figure 2B). No statistically significant increase of good clinical outcomes (43.06% vs 41.78%; OR=1.14; 95% CI, 0.77 to 1.69; P=0.52;) or excellent clinical outcomes were found following endovascular therapy (30.43% vs 30.42%; OR=1.05; 95% CI, 0.80 to 1.38; P=0.72;) (Figure 2).

### Secondary Outcomes

#### Symptomatic hemorrhage

**Figure 3:**
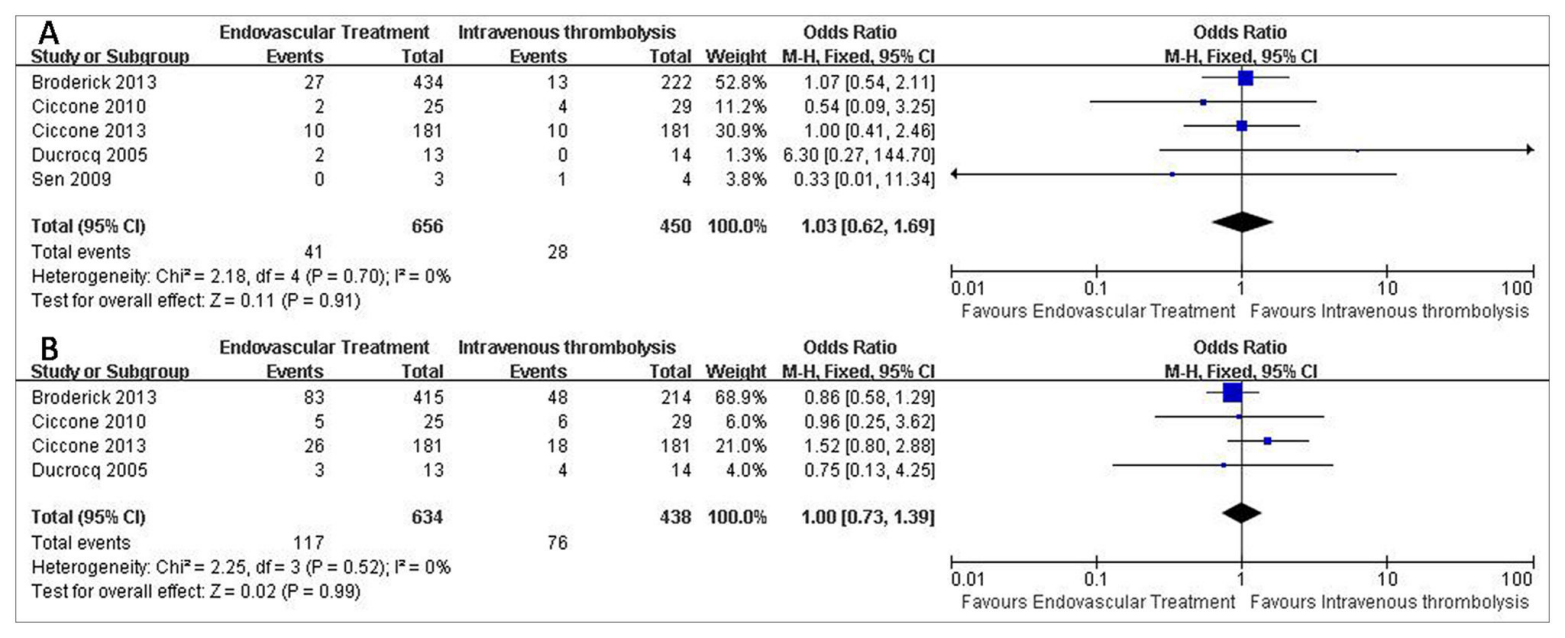
Secondary Outcomes of patients treated with Endovascular Therapy and Intravenous Thrombolysis after acute ischemic stroke. (A) Forest plot of RR and 95% CI for the occurrence of symptomatic hemorrhage in patients assigned to Endovascular Therapy and Intravenous Thrombolysis. (B) Forest plot of RR and 95% CI for the occurrence of mortality in patients assigned to Endovascular Therapy and Intravenous Thrombolysis.

Data for symptomatic hemorrhage were available from five trials, representing 1106 patients (Figure 3A). A symptomatic hemorrhage of 6.25% was noticed in the endovascular therapy group compared with 6.22% in the intravenous thrombolysis group. The corresponding pooled OR was 1.03 (95% CI, 0.62 to 1.69; P=0.91;).

#### All-Cause Mortality

Four studies presented data on all-cause mortality (Figure 3B). A mortality of 18.45% was noted in the endovascular therapy group compared with 17.35% in the intravenous thrombolysis group. We used the Mantel-Haenszel fixed-effects model. The pooled OR was 1.00(95% CI, 0.73 to 1.39; P=0.99;). 

**Figure 4:**
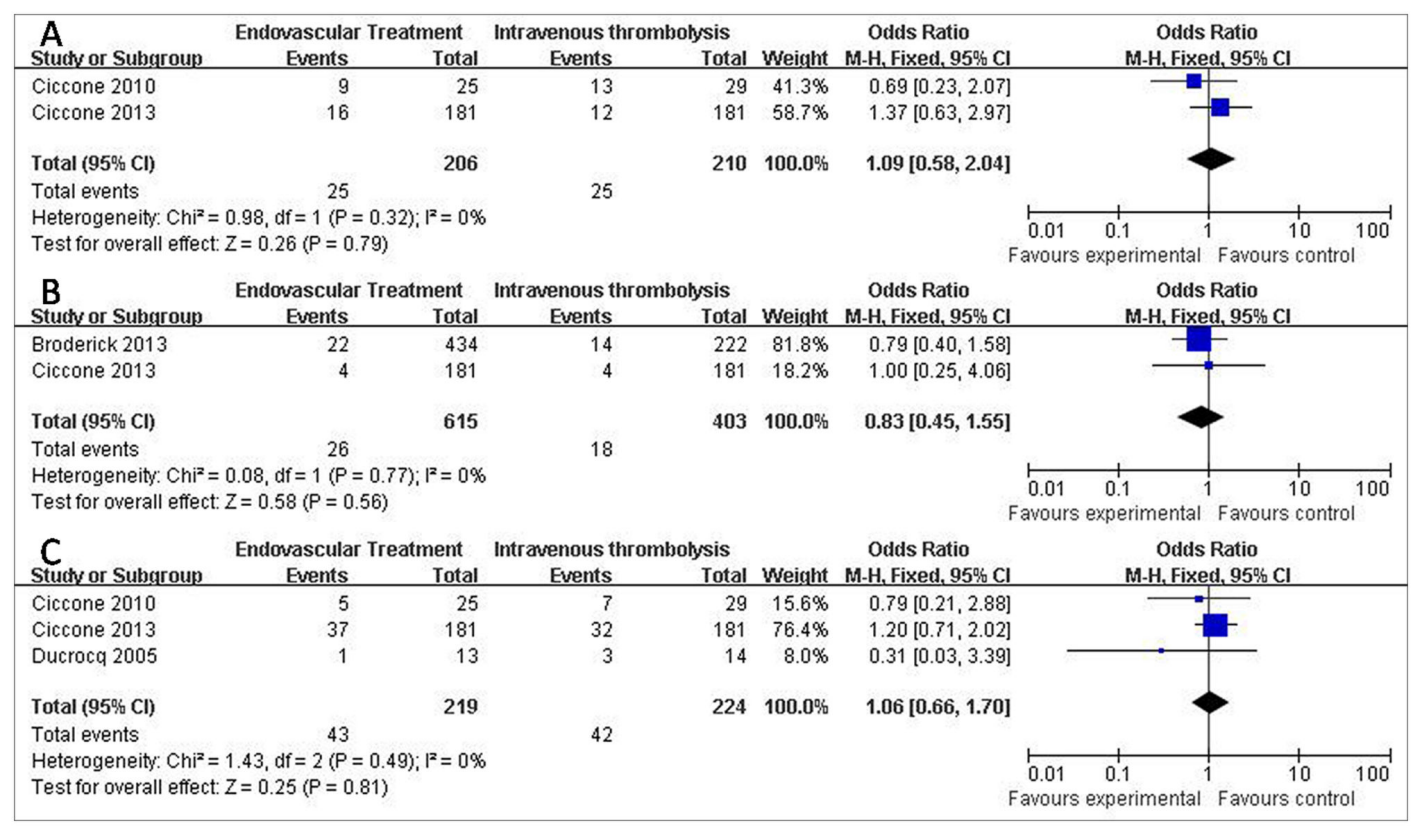
Other adverse effects of patients treated with Endovascular Therapy and Intravenous Thrombolysis after acute ischemic stroke. (A) Forest plot of RR and 95% CI for the occurrence of Neurologic deterioration at Day 7 in patients assigned to Endovascular Therapy and Intravenous Thrombolysis. (B) Forest plot of RR and 95% CI for the occurrence of recurrent ischemic stroke in patients assigned to Endovascular Therapy and Intravenous Thrombolysis. (C) Forest plot of RR and 95% CI for the occurrence of cerebral edema in patients assigned to Endovascular Therapy and Intravenous Thrombolysis.

For additional end points, there was no significant difference in neurologic deterioration at Day 7 (12.14% vs 11.90%; OR = 1.09; 95% CI, 0.58 to 2.04; P=0.79),recurrent ischemic stroke (4.23% vs 4.47%; OR = 0,83; 95% CI, 0.45 to 1.55; P=0.56), and cerebral edema (19.63% vs 18.75%; OR = 1.06; 95% CI, 0.66 to 1.70; P=0.81) between tow groups (Figure 4).

### Analysis for Publication Bias and Sensitivity

In our assessment of the funnel plot of each meta-analysis, no evidence for publication bias was indicated. The sensitivity analysis using the fixed-effects model yielded estimates similar to those of the random-effects model for the risk of good and excellent outcomes (RR 1.07, 95% CI 0.83–1.37, P=0.61;I^2^= 41%; RR 1.03, 95% CI 0.85–1.24, P=0.78,I^2^= 41%). The sensitivity analysis using the random-effects model also yielded estimates similar to those of the fixed-effects model for the risk of symptomatic hemorrhage and mortality (RR 1.01, 95% CI 0.61–1.68, P=0.96,I^2^= 0%; RR 1.00, 95% CI 0.72–1.38, P=1.00,I^2^= 0%).

## Discussion

Intravenous thrombolysis is the standard treatment for acute ischemic stroke, but its clinical effectiveness is critically time-dependent [5],[6]. Endovascular treatment as alternative treatments has been used for many years. Current endovascular approaches include endovascular pharmacologic thrombolysis, manipulation of the clot with the use of a guidewire or microcatheter, mechanical and aspiration thrombectomy, and most recently, stent-retriever technology. But it is not known whether clinical outcomes are superior with endovascular therapy as compared with the standard therapy. In this current meta-analysis including 1106 patients hospitalized for acute ischemic stroke, we identified 5 RCTs evaluating the efficacy and safety of endovascular treatment on different outcome measures.

In included trials, only one that used urokinase compared IAT with IVT [18]. Others used recombinant tissue plasminogen activator (alteplase) or tissue plasminogen activator (t-PA) [9],[10][19],,[20]. They are all recommended thrombolytic drugs for acute ischemic stroke treatment [7],[21][22].

The high rate of recanalization with endovascular treatment might give the impression that this method is effective in most cases, although it may provide no clinical benefit in almost half the patients [23]. Rather than depending on the route of administration of thrombolytics, the rates of recanalization are influenced by the occluded segment of the artery; the distal, typically embolic occlusions recanalize more easily than the more proximal ones. Recanalization and restoration of nutritive perfusion are the presumed main mechanisms of thrombolytic treatment [24].

In our meta-analysis, beneficial effects of endovascular treatment were not statistically significant for increased rates of both good and excellent final clinical outcomes between tow groups. Endovascular treatment usually need more time than intravenous thrombolysis. In some patients, the ischemic area is already fully infarcted before intervention, with no penumbral tissue remaining that reperfusion can salvage [25]. Efficacy is strongly related to both the rapidity of initiation of treatment and the severity and extent of ischemia [26][27]. Maybe minimization of the time to treatment will produce different results. 

Prior studies suggest that many postfibrinolysis hemoorhage are confined to already-damaged tissue and do not worsen final outcome [28][29]. In our meta-analysis, Safety of endovascular treatment was not statistically significant for increased rates of symptomatic hemorrhage or mortality. For additional end points, there was no significant difference in neurologic deterioration at Day 7, recurrent ischemic stroke, and cerebral edema between tow groups. 

In the included trials, only one RCT compared a combined approach (intravenous t-PA followed by endovascular therapy) with intravenous t-PA alone [9]. The trial failed to show a benefit in functional outcomes with the use of additional endovascular therapy, as compared with the standard therapy of intravenous t-PA alone [9].

Our study has some limitations. Endovascular therapy as compared with intravenous t-PA often need more time. Minimization of the time to treatment will be essential for assessing the potential benefit of endovascular therapy for acute ischemic stroke.

Device technology is advancing rapidly, and it is conceivable that the latest-generation devices, which were used infrequently in this article, could provide greater benefit if used widely specially for larger artery occlusion [30][31]. Stent retrievers were used in only a small number of patients. Hence, the other limitation of our article is that it did not compare the efficacy of the new stent retrievers with that of intravenous t-PA alone.

### Conclusions

In conclusion, formal meta-analysis suggests that endovascular therapy may produce similar good and excellent clinical outcomes, symptomatic hemorrhage and mortality as compared with intravenous thrombolysis in acute ischemic stroke. The use of the more invasive and expensive endovascular therapy for acute ischemic stroke may be not necessary. 

## Supporting Information

Checklist S1 **The PRISMA checklist of the manuscript.**
(DOC)
